# Effect of SNPs in HSP Family Genes, Variation in the mRNA and Intracellular Hsp Levels in COPD Secondary to Tobacco Smoking and Biomass-Burning Smoke

**DOI:** 10.3389/fgene.2019.01307

**Published:** 2020-01-09

**Authors:** Enrique Ambrocio-Ortiz, Gloria Pérez-Rubio, Alejandra Ramírez-Venegas, Rafael Hernández-Zenteno, Alma D. Del Angel-Pablo, Martha E. Pérez-Rodríguez, Ana M. Salazar, Edgar Abarca-Rojano, Ramcés Falfán-Valencia

**Affiliations:** ^1^ HLA Laboratory, Instituto Nacional de Enfermedades Respiratorias Ismael Cosío Villegas, Mexico City, Mexico; ^2^ Tobacco Smoking and COPD Research Department, Instituto Nacional de Enfermedades Respiratorias Ismael Cosío Villegas, Mexico City, Mexico; ^3^ Unit of Medical Research in Immunology CMN S-XXI, Instituto Mexicano del Seguro Social, Mexico City, Mexico; ^4^ Department of Genomic Medicine and Environmental Toxicology, Instituto de Investigaciones Biomédicas, Universidad Nacional Autónoma de México, Mexico City, Mexico; ^5^ Sección de Estudios de Posgrado e Investigación, Escuela Superior de Medicina, Instituto Politécnico Nacional, Mexico City, Mexico

**Keywords:** chronic obstructive pulmonary disease, HSPA1A, genetic susceptibility, biomass-burning smoke, Hsp70

## Abstract

Heat shock proteins (HSP) genes are a superfamily responsible for encoding highly conserved proteins that are important for antigen presentation, immune response regulation, and cellular housekeeping processes. These proteins can be increased by cellular stress related to pollution, for example, smoke from biomass burning and/or tobacco smoking. Single nucleotide polymorphisms (SNPs) in these genes could affect the levels of their proteins, as well as the susceptibility to developing lung diseases, such as chronic obstructive pulmonary disease (COPD), related to the exposure to environmental factors.

**Methods:** The subjects included were organized into two comparison groups: 1,103 smokers (COPD patients, COPD-S = 360; smokers without COPD, SWOC = 743) and 442 never-smokers who were chronically exposed to biomass smoke (COPD patients, COPD-BS = 244; exposed without COPD, BBES = 198). Eight SNPs in three *HSP* genes were selected and genotyped: four in *HSPA1A*, two in *HSPA1B*, and two in *HSPA1L*. Sputum expectoration was induced to obtain pulmonary cells and relative quantification of mRNA expression. Subsequently, the intracellular protein levels of total Hsp27, phosphorylated Hsp27 (Hsp27p), Hsp60, and Hsp70 were measured in a sample of 148 individuals selected based on genotypes.

**Results:** In the smokers’ group, by a dominant model analysis, we found associations between rs1008438 (CA+AA; p = 0.006, OR = 1.52), rs6457452 (CT+TT; p = 0.000015, OR = 1.99), and rs2763979 (CT+TT; p = 0.007, OR = 1.60) and the risk to COPD. Among those exposed to biomass-burning smoke, only rs1008438 (CA+AA; p < 0.01, OR = 2.84) was associated. Additionally, rs1008438 was associated with disease severity in the COPD-S group (AA; p = 0.02, OR = 2.09). An increase in the relative expression level of *HSPA1A* was found (12-fold change) in the COPD-BS over the BBES group. Differences in Hsp27 and Hsp60 proteins levels were found (p < 0.05) in the comparison of COPD-S vs. SWOC. Among biomass-burning smoke-exposed subjects, differences in the levels of all proteins (p < 0.05) were detected.

**Conclusion:** SNPs in HSP genes are associated with the risk of COPD and severe forms of the disease. Differences in the intracellular Hsp levels are altered depending on the exposition source.

## Introduction

Chronic obstructive pulmonary disease (COPD) is a preventable and treatable disease; its main characteristic is progressive respiratory airways obstruction related to a chronic inflammatory response against noxious particles [Global Initiative for Chronic Obstructive Lung Disease (GOLD), 2013; Montes de Oca et al., 2015]. Worldwide, COPD has an ~10% prevalence. In 2012, approximately 6% of deaths were caused by COPD, a high proportion of which were in low- and middle-income countries (World Health Organization, N., 2008; OMS | Enfermedad pulmonar obstructiva crónica (EPOC), 2015). Different risk factors have been described, including environmental and intrinsic ones [Menezes et al., 2005; Global Initiative for Chronic Obstructive Lung Disease (GOLD), 2013]. The environmental risk factors include smoking (OMS | Tabaco, 2015) and chronic exposure to biomass-burning smoke (BBS) (Raj, 2014), while all the characteristics of the patients themselves are considered intrinsic factors (age, sex, individual genetic constitution) [Sørheim et al., 2010; Global Initiative for Chronic Obstructive Lung Disease (GOLD), 2013]. Approximately 10 million people worldwide are smokers, and 80% live in emergent countries. In 2017, the national addiction survey (*Encuesta Nacional de Adicciones*, *ENCODAT*) reported that in Mexico, 22% of the population are current smokers (Salud, 2017). There are no reports or monitoring of BBS, so the information about this risk factor is limited. It is known that ~30% of COPD cases are secondary to BBS exposure, with women being more affected than men (Ramírez-Venegas et al., 2006; Camp et al., 2014).

In cases caused by intrinsic factors, women are most susceptible to severe forms of the disease (Perez-Padilla et al., 1996). Several biological pathways have been studied looking for genetic polymorphisms that could affect the susceptibility to the illness, as well as the severity (Chen et al., 2015; Gharib et al., 2015). Some of these polymorphisms are located in genes related to inflammation, oxidative stress, and cellular death processes (Yanbaeva et al., 2009; Brzóska et al., 2014; Reséndiz-Hernández et al., 2015). One example is the heat shock proteins (HSP) family, a group of genes that encode a great number of highly conserved proteins called Hsp. These proteins have a role in protein degradation and protein-folding, and hormonal and corticosteroid receptor activator/stabilizers can be detected as damage signals and even help in antigen presentation (Javid et al., 2007; Soo et al., 2008; Dokladny et al., 2010; Hodge et al., 2016). Although Hsp level variation has been described as clinically and biochemically relevant (Jain et al., 2014; Hodge et al., 2016), the effects of genetic variation on the expression of the proteins or the susceptibility to COPD secondary to tobacco smoke or BBS remain unclear.

Based on the above, we designed a genetic association study between SNPs in *HSPA1A*, *HSPA1B*, and *HSPA1L* and the susceptibility to COPD secondary to tobacco smoke and BBS, specifically, the effect on COPD susceptibility and severity in Mexican mestizo patients, as well as the effect on these genes’ mRNA and protein levels in lung cells.

## Materials and Methods

### Study Population

The present case-control study included 1,545 subjects who went to the clinic for treatment of COPD and/or to the support clinic for guidance in smoking cessation. Both these services are part of the Department of Smoking and COPD Research Department of the Instituto Nacional de Enfermedades Respiratorias Ismael Cosio Villegas (INER), in Mexico City.

Participants in this study were classified into two comparison groups. The first group included patients with COPD secondary to smoking (COPD-S) and smokers without the illness (SWOC), in both cases with tobacco index (TI) ≥5 (≥10 cigarettes per day, ≥10 years smoking). In the second comparison group, patients with COPD secondary to BBS (COPD-BS) and BBS-exposed subjects without the disease (BBES), both with biomass-smoke exposure index (BSEI) ≥100 h/year and never-smokers. BBS-exposed subjects were part of the National Program for equality between women and men with the COPD timely diagnostic campaign in women of rural populations, mainly in Oaxaca’s northern highlands and suburban areas in the Tlalpan mayoralty of Mexico City (Ramírez-Venegas et al., 2018). Specialized chest physicians using the diagnosis and severity criteria described in the GOLD guidelines completed the diagnosis and clinical evaluation. The Strengthening the Reporting of Genetic Association Studies (STREGA) guidelines were taken into account in the design of this genetic association study (Little et al., 2009).

All participants filled out a hereditary pathology background survey. Exclusion criteria included non-Mexican ancestry and chronic respiratory diseases other than COPD and/or inflammatory disorders. Patients who met the inclusion criteria were invited to participate after being given a detailed description of the study. All participants signed an informed consent form and were provided with a privacy statement describing the protection of personal data. The Ethics in Research Committee of the INER in Mexico City reviewed and approved the current protocol (protocol numbers B14-17 and B11-19).

### DNA Samples

The processing of whole-blood samples began with centrifugation for 8 min at 4,500 rpm to separate the plasma, which was stored in cryopreservation tubes at -80°C until use. Genetic material was extracted from the cell pellet with the commercial BDtract DNA isolation kit (Maxim Biotech, San Francisco, CA, USA) and then rehydrated in TE buffer (Ambion, Waltham MA, USA). Subsequently, the extracted material was quantified using a Nanodrop 2000 device (Thermo Scientific, Wilmington, DE, USA) and stored at -80°C until further processing.

### SNP Genotyping

SNPs included in the study were chosen from previous reports on other respiratory diseases and based on a minor allele frequency (MAF) > 10% in the 1,000 Genomes Project (**Table 1**). rs2227956, despite not complying with these criteria, was included due to its position in the *HSPA1L* gene.

**Table 1:** Molecular characteristics of SNPs included in this study.

Genes	SNP	Position	Change	MAF
*HSPA1A*	rs562047	Exon	G/C	0.18^1^
	rs1008438	Promoter	C/A	0.49^1^
	rs1043618	Promoter	G/C	0.37^1^
	rs1061581	Exon	G/A	0.33^2^
*HSPA1B*	rs6457452	Promoter	C/T	0.099^1^
	rs2763979	Promoter	C/T	0.42^1^
*HSPA1L*	rs17856061	Promoter	C/G	0.18^1^
	rs2227956	Exon	T/G	0.04^3^

^1^ 1000 Genomes Project, Hispanic population.

^2^ EGP (Environmental Genome Project) SNPs for the Hispanic population.

^3^ HapMap for the Mexican population.

### Genotype and Allele Discrimination

Genotyping was performed using TaqMan allele-discrimination real-time PCR with predesigned probes in a 7300 Real-Time PCR System (Applied Biosystems, Foster City CA, USA). Genotype assignment was performed based on allele discrimination and was confirmed by absolute quantitation. In addition, three wells without template (contamination controls) were included for each genotyping plate, and 1% of the samples included in the study were genotyped in duplicate for control allele assignment.

The amplification mix was prepared with the corresponding TaqMan probe (Applied Biosystems, CA, USA), *TaqMan*™ *Universal PCR Master Mix* (Applied Biosystems; Woolston, UK), and nuclease-free water (Maxim Biotech, San Francisco, CA, USA) to adjust the final reaction volume. Five microliters of the amplification mix and 3 µl of adjusted DNA were put into *MicroAmp^®^ Optical 96-well Reaction Plates* (Applied Biosystems; Woolston, UK), with *MicroAmp^®^ Optical Adhesive Film* (Applied Biosystems; Woolston, UK) to cover the plates. TaqMan fluorophores VIC and FAM were added to mark probes and identify the different alleles. The amplification was carried out in the *7300 PCR real-time system* (Applied Biosystems; Woolston, UK) following the supplier’s indications. Data were interpreted using the Sequence Detection Software (SDS v. 1.4, Applied Biosystems).

### Sputum Induction and Cell Purification

Based on genotypes, we selected a subsample of participants for deeper characterization. To obtain sputum, participants were nebulized with a sterile 7% saline solution. Treatment lasted for 5 min, followed by a rest period of 5 min. Treatment and rest cycles were repeated three times. The sample was mechanically disaggregated using phosphate buffered saline (PBS) buffer 1X (Invitrogen; Carlsbad, CA, USA) in equal volumes to eliminate excess mucus, followed by centrifugation for 10 min at 3,000 rpm. The supernatant was discarded, and another lavage was made with 20 ml of buffer PBS, followed by centrifugation for 5 min at 3,000 rpm. The cellular pellet was resuspended in 10 ml of PBS and centrifuged for 5 min at 3,000 rpm. The PBS 1X was decanted, and the cells were stored in RPMI 1640 medium (Life Technologies Co. Carlsbad, CA, USA), with fetal bovine serum (Life Technologies Co. Carlsbad, CA, USA) at 10% and dimethyl sulfoxide (DMSO) (Sigma; Saint-Quentin Fallavier, France) at 10%.

### mRNA Extraction and cDNA Synthesis

RNA extraction was carried out by the TRIzol (Sigma-Aldrich, Co. St Louis, MO, USA)/chloroform (IBI Scientific, Kapp Court Peosta, IA. USA) method. The mRNA obtained was rehydrated with 50 µl of nuclease-free water (Maxim Biotech, San Francisco, CA, USA). Quantification was carried out by UV-vis spectrophotometry at 260 nm using a Nanodrop 2000 (*Thermo Scientific;* Wilmington, DE, USA). mRNA integrity was checked by 2% agarose gel electrophoresis. For cDNA synthesis, we used the RevertAid™ cDNA first-strand synthesis kit (*Thermo Scientific;* Lithuania) following the supplier’s specifications. cDNA samples were stored at -80°C until use.

### Gene Relative Expression Levels

Fifteen cDNA samples from each study group were chosen based on the genotyping results obtained from the genetic association study. We used commercial TaqMan™ (*Applied Biosystems*, CA, USA) for genetic expression (Hs00359163_s1 for *HSPA1A*, Hs01040501_sH for *HSPA1B*, Hs00271466_s1 for *HSPA1L*), Universal Master Mix II with UNG (*Applied Biosystems, CA*, USA) and nuclease-free water. The *GAPDH* gene was chosen as the endogenic control for data normalization. All samples were amplified in triplicate, including *GAPDH*. The 2^-ΔΔCT^ method was used to calculate relative expression levels between cases and controls. In order to corroborate the effect of the SNPs associated, we carried out a Quantitative Trait Association (QTA) analysis using the relative expression data and the genotyping data applying Wald test.

### Hsp Protein Levels Quantification

A sample of 176 subjects was chosen: 88 smokers (COPD-S = 44, SWOC = 44) and 88 BBS exposed (COPD-BS = 44, BBES = 44). Blood-EDTA tube samples were centrifuged for 8 min at 4,500 rpm to separate plasma from peripheral blood mononuclear cells (PBMCs). PBMCs were isolated by centrifugation in a Ficoll-Hypaque Lymphoprep (Stemcell Technologies, Melbourne, Australia) density gradient. Cells were washed with PBS 1X and stored in RPMI medium supplemented with fetal bovine serum and DMSO (Sigma; Saint-Quentin Fallavier, France) at 10% each.

### Intracellular Hsp Quantification

For cellular lysis and Hsp quantification, the commercial 5-plex heat shock protein magnetic bead kit (Cat. 48-615MAG Millipore, Missouri, USA) was used to detect Hsp27, phosphorylated Hsp27 (Hsp27p), and Hsp60, Hsp70. Before lysis, PBMCs were washed with PBS 1X to eliminate any compound that could interfere in the analysis. At the same time, a cOmplete Protease Inhibitor Cocktail (Cat. 04693116001 Roche, Mannheim, Germany) tablet was added to the lysis buffer included in the kit to avoid degradation of the protein of interest. Cell lysis was quantified by the bicinchoninic acid assay (BCA) method using the Pierce BCA Protein Assay Kit (Thermo Scientific; Lithuania) and Nanodrop 2000 device. Samples were homogenized and adjusted for further quantification in the Luminex^®^ LABScan 100 (Luminex Corp. Austin TX, USA) system.

### Statistical Analysis

Adherence to Hardy-Weinberg equilibrium (HWE) was confirmed with Finetti v.3.0.8 software. Genotype analysis was carried out with Pearson’s chi-squared and Fisher’s exact tests using Epi Info v. 7.1 software (Epi Info™ | CDC, 2015). To adjusted significance values, Bonferroni correction (multiple testing) was applied to the association results. The population and pulmonary function data, plasma determinations, and all correlations were analyzed with SPSS v. 24. The examination of a possible association by genotype was performed with Epidat version 3.1 software [Public Health Information Department of the General Public Health Administration of the Health Consulate (Directorate of Galicia) and (OPS-WHO), 2006], using simple 2×3 contingency tables, a codominant model, and a 95% confidence level. In addition, the software PLINK 1.07 (Purcell et al., 2007) was used, and a logistic regression model (1 degree of freedom) was created including sex, age, and TI as co-variables in the smokers’ group; in the BBES groups only age and BSEI were included. Correlation between SNPs and relative expression levels was evaluated by QTA applying Wald test.

## Results

### Population Description

We included 1,545 subjects in two comparisons; the first included 1,103 smokers, 360 with COPD (COPD-S) and 743 without the illness (SWOC). In the second comparison, 442 subjects with chronic BBS exposure (BBE) were included. Of these, 244 were patients with COPD (COPD-BS) and 198 were subjects without COPD (BBES) who had never smoked and lacked clinical symptoms, radiological, or physiological alterations in lung function (**Table 2**).

**Table 2:** Demographic and lung function data.

	COPD-S (n = 360)	SWOC (n = 743)	p-value	COPD-BS (n = 244)	BBES (n = 198)	p-value
Age (years)	68 (62.0–75.0)	53 (47.0–59.3)	<0.01	74.0 (67.0–79.0)	61.0 (53.5–68.5)	<0.01
M/F (%)	76.9/23.1	50.7/49.3	<0.01	13.8/86.2	17.8/82.2	0.31
BMI	27.1 (24.4–30.1)	25.5 (22.5–28.4)	0.18	26.5 (23.2–31.2)	27.6 (24.5–30.7)	0.28
**Smoking and biomass-smoke exposition index**						
TI (packs/year)	40.0 (28.0–40.0)	28 (19.0–40.0)	<0.01	NA	NA	NA
BSEI (h/year)	NA	NA	NA	240.0 (160.0–421.0)	160.0 (120.0–240.0)	<0.01
**Lung function values (post)**						
FVC%	55.5 (38.0–75.0)	97.0 (87.0–108.0)	<0.01	60.0 (51.0–78.0)	96.0 (81.5–109.0)	<0.01
FEV_1_%	85.5 (70.0–101.0)	95.0 (85.0–105.0)	<0.01	85.0 (69.0–99.0)	93.0 (79.0–105.0)	<0.01
FEV_1_/FVC	53.11 (38.5–63.6)	82.2 (78.0–86.0)	<0.01	53.5 (46.3–67.0)	96.0 (84.0–106.0)	<0.01

Data are presented as a function of the median interquartile range (25–75). p-values were calculated by the Mann-Whitney-U test. We considered p < 0.05 to indicate significance.

In the smoker comparison, we found a difference in age (COPD-S 68 years *vs.* SWOC 53 years). There was also a difference in sex, with a greater presence of males, especially in the COPD-S group (76.9% *vs.* 50.7%, < 0.01). Additionally, the tobacco index (TI) was greater in the COPD-S group (TI = 40) compared with SWOC (TI = 28). No differences in BMI were found.

In the BBS-exposed subjects, we found a difference in the median age (COPD-BS = 74 years, BBES = 61 years). There was a predominant presence of women (>80%) in both groups but without significant differences. The BSEI was greater in the COPD-BS group (240 h/year) than in the BBES group (160 h/year). In both groups, we found differences in the FEV_1_, FVC, and FEV_1_/FVC data values. For this reason, we considered age, sex, TI, and BSEI as covariables for statistical data analysis.

### Hardy-Weinberg Equilibrium (HWE)

We found HWE deviation (p < 0.05) in four out of eight SNPs included in the study (rs6457452, rs2763979, rs17856061, and rs2227956) in both control groups (**Table 3**). For this reason, we only considered relevant the results observed in rs562047, rs1008438, rs1043618, and rs1061581.

**Table 3:** Hardy-Weinberg equilibrium.

**SNP**	**SWOC**		**p-value**	**BBES**		**p-value**
	**Obs. Het.**	**Pred. Het.**		**Obs. Het.**	**Pred. Het.**	
rs562047	0.243	0.271	0.269	0.319	0.337	0.061
rs1008438	0.498	0.492	0.961	0.509	0.462	0.247
rs1043618	0.472	0.451	0.391	0.463	0.438	0.605
rs1061581	0.512	0.5	0.456	0.527	0.499	0.884
rs6457452	0.764	0.486	<0.01	0.775	0.483	<0.01
rs2763979	0.571	0.499	<0.01	0.566	0.499	0.032
rs17856061	0.229	0.272	<0.01	0.363	0.272	0.016
rs2227956	0.818	0.483	<0.01	0.783	0.483	<0.01

Data of all the SNPs included in the study, as well as the heterozygote proportions. We present p values for Hardy-Weinberg equilibrium in both control groups. Obs. Het., observed heterogeneity; Pred. Het., predicted heterogeneity; MAF, minor allele frequency.

### Genetic Association Analysis

Among smokers, there were significant differences in the allele frequencies of six out eight SNPs analyzed (rs562047 and rs1008438 in *HSPA1A*; rs6457452 and rs2763979 in *HSPA1B*; rs17856061 and rs2227956 in *HSPA1L*). There was a MAF increase in the case group, except for rs2227956, where controls had a higher MAF. After Bonferroni correction, only three out eight SNPs remained associated (rs562047, p < 0.01; rs2763979, p = 0.001; rs1008438, p = 0.02) (**Supplementary Table 1**). Alleles and genotypes were analyzed after adjustment by logistic regression using the covariables mentioned above; for the smoking groups, we included sex, age, and TI. The associations were conserved only for rs1008438 (p < 0.01, OR = 1.48), rs562047 (p < 0.01, OR = 2.31), rs1061581 (p = 0.02, OR = 0.74), and rs2763979 (p < 0.1, OR = 1.69).

Comparing genotypes under the codominant model, we found rs562047 (p = 0.01, GC = 1.68), rs1008438 (p < 0.01 CA OR = 1.41, AA OR = 1.83), rs6457452 (p < 0.01; CT, OR = 1.89. TT, OR = 2.85), rs2763979 (p < 0.01, CT OR = 1.46, TT = 2.06), and rs17856061 (p < 0.01, CG OR = 2.93) were associated with risk of COPD, while rs2227956 (p < 0.01, TG OR = 0.39) was associated with decreased risk of the illness. Odds ratio values for the associated SNPs were higher in the minor allele homozygous state, except for rs562047 and rs17856061 (OR of CG > GG) (**Supplementary Table 2**).

Based on the findings with the codominant model and only considering the SNPs that met HWE, we decided to use a dominant model to corroborate the findings for rs1008438, rs6457452, and rs2763979. Under the dominant model, we corroborated the findings that rs1008438, rs6457452, and rs2763979 were associated with an increased risk of COPD (**Table 4**).

**Table 4:** Genotype comparison by dominant model, COPD-S *vs.* SWOC.

Gene/SNP	COPD-S	SWOC	p-value	OR	CI (95%)
	n = 360 (%)	n = 743 (%)			
***HSPA1A***					
rs1008438 (C/A)					
CC	25.76	34.49	0.006	0.65	0.48–0.87
CA+AA	74.24	65.51		1.52	1.13–2.03
***HSPA1B***					
rs6457452 (T/C)					
CC	13.78	24.20	0.000015	0.50	0.35–0.71
CT+TT	86.22	75.80		1.99	1.40–2.85
rs2763979 (C/T)					
CC	14.37	21.41	0.007	0.62	0.44–0.87
CT+TT	85.63	78.59		1.60	1.15–2.29

p-values were calculated by Fisher's exact test. Associations were considered significant when p < 0.05.

Among those exposed to BBS, the allele frequencies of the COPD-BS and BBES groups showed significant differences at rs1008438 (A allele, p < 0.01) and rs17856061 (C allele, p = 0.01). The alleles at the other SNPs did not show significant differences in their frequencies. We ran a logistic regression including sex and BSEI since there were significant differences. After this adjustment, only rs1008438 (p = 0.01, OR = 1.6) and rs1061581 (p < 0.01, OR = 0.53) remained significant (**Supplementary Table 3**).

Genotype frequencies were compared under a codominant model. We found associations between rs1008438 (AA, p < 0.01, OR = 1.43), rs1043618 (CC, p < 0.01, OR = 0.23), and rs17856061 (GG, p = 0.02, OR = 0.47) and COPD (**Table 5**). These findings were checked under the dominant model, and just rs1008438 in *HSPA1A* maintained its association with the disease (p < 0.01 CA+AA OR = 2.84) (**Supplementary Table 4**).

**Table 5:** Genotype comparison in BBES subjects under the codominant model.

Gen/SNP	COPD-BS	BBES	p-value	OR	CI (95%)
	n = 244 (%)	n = 198 (%)			
***HSPA1A***					
rs562047					
GG	62.24	65.50	1.0 (Ref.)		
GC	31.95	26.00	0.92	1.12	(0.93–1.34)
CC	5.81	8.50		0.85	(0.57–1.27)
rs1008438					
CC	29.24	38.78	1.0 (Ref.)		
CA	50.85	50.00	<0.01	1.16	(0.94–1.43)
AA	19.92	11.22		1.43	(1.13–1.81)
rs1043618					
GG	44.67	44.90	1.0 (Ref.)		
GC	46.31	45.41	<0.01	0.23	(0.14–0.39)
CC	9.02	9.69		0.97	(0.71–1.32)
rs1061581					
GG	25.71	25.26	1.0 (Ref.)		
GA	52.65	50.52	0.81	1.01	(0.83–1.23)
AA	21.63	24.23		0.94	(0.74–1.21)
***HSPA1B***					
rs6457452					
CC	17.68	21.18	1.00 (Ref.)		
CT	77.53	75.95	0.79	0.98	(0.81–1.29)
TT	4.79	2.87		1.02	(0.92–1.15)
rs2763979					
CC	14.37	21.41	1.00 (Ref.)		
CT	56.62	57.60	0.61	1.03	(0.85–1.25)
TT	29.01	20.99		0.94	(0.84–1.34)
***HSPA1L***					
rs17856061					
CC	58.54	72.53	1.00 (Ref.)		
CG	36.13	22.53	0.02	0.98	(0.80–1.19)
GG	5.32	4.95		0.47	(0.24–0.89)
rs2227956					
TT	21.67	18.46	1.00 (Ref.)		
TG	78.33	81.40	0.73	0.95	(0.78–1.16)
GG	0.00	0.13		1.14	(0.50–2.59)

p-values were calculated by Fisher's exact test. Associations were considered significant when p < 0.05.

### SNPs and COPD Severity

Cases from the COPD-S and COPD-BS groups were classified according to GOLD stage in groups of less severity as G1 (GOLD1 + GOLD2) and greater severity as G2 (GOLD3 + GOLD4). rs1008438 was found to be associated with the development of severe forms of the illness in the COPD-S group (p = 0.02, AA OR = 2.09). No association in the COPD-BS group was detected (**Table 6**).

**Table 6:** Genotype comparison stratifying by severity in the COPD-S group.

Gene/SNP	G2 FG%	G1 FG%	p-value	OR	CI (95%)
	AF% (n = 93)	AF% (n = 190)			
***HSPA1A***					
rs562047					
GG	66.67	63.69	0.74	1.13	0.65–1.97
GC	22.58	31.52	0.17	0.63	0.34–1.15
CC	10.75	4.79	0.13	2.39	0.87–6.52
rs1008438					
CC	22.48	27.54	0.48	0.76	0.40–1.42
CA	42.69	52.17	0.21	0.68	0.39–1.16
AA	34.83	20.29	0.02	2.09	1.15–3.83
rs1043618					
GG	43.48	35.81	0.27	1.38	0.81–2.34
GC	45.65	54.05	0.25	0.71	0.42–1.20
CC	10.87	10.14	0.97	1.08	0.46–2.52
rs1061581					
GG	24.44	17.81	0.29	1.49	0.78–2.83
GA	54.44	58.22	0.66	0.86	0.50–1.45
AA	21.12	23.97	0.73	0.85	0.45–1.59
***HSPA1B***					
rs6457452					
CC	12.50	15.00	0.58	0.77	0.36–1.72
CT	85.23	81.43	0.41	1.43	0.70–2.92
TT	2.27	3.57	0.69	0.41	0.04–3.57
rs2763979					
CC	15.22	13.61	0.73	1.19	0.58–2.41
CT	54.35	57.14	0.59	0.85	0.51–1.39
TT	30.43	29.25	0.80	1.11	0.64–1.90
***HSPA1L***					
rs17856061					
CC	64.52	54.36	0.15	1.52	0.89–2.60
CG	25.81	40.27	0.03	0.51	0.29–0.91
GG	9.68	5.37	0.31	1.89	0.70–5.08
rs2227956					
TT	22.58	20.13	0.61	1.16	0.62–2.17
TG	77.42	79.87	0.74	0.86	0.46–1.62
GG	0.00	0.00	———	———	———

p-values were calculated by Fisher's exact test. Associations were considered significant when p < 0.05.

### Gene Expression Assays

Relative gene expression levels in the study groups were normalized using *GAPDH* as an endogenous control. In COPD-S *vs.* SWOC, we observed a reduction in expression levels in the three genes (*HSPA1A*_2^-ΔΔCT_ = 1.07, *HSPA1B*_2^-ΔΔCT_ = 0.29 and *HSPA1B*_2^-ΔΔCT_ = 0.44). *HSPA1A* had the smallest reduction among the three genes. Interestingly, in the COPD-BS *vs.* BBES comparison, we found an increase in the levels of the three genes (*HSPA1A*_2^-ΔΔCT_ = 12.1, *HSPA1B*_2^-ΔΔCT_ = 1.6 and *HSPA1B*_2^-ΔΔCT_ = 9.2). *HSPA1A* showed a marked increase in the relative expression level among all analyzed genes (**Table 7**). QTA analysis was made between *HSPA1A* mRNA and rs1008438. We did not find an association between the relative expression levels and the SNP.

**Table 7:** *HSP* relative expression levels in comparison groups.

	ΔCT		
	*_HSPA1A-GAPDH_*	*_HSPA1B-GAPDH_*	*_HSPA1L-GAPDH_*
*COPD-P*	8.0±1.4	9.8±3.2	9.7±.1.2
*SWOC*	8.1±2.2	8.1±1.2	8.6±1.2
ΔΔCT *_COPD-S-SWOC_*	-0.1	1.7	1.1
2^-ΔΔCT *_COPD-S-SWOC_*	1.07	0.29	0.44
*COPD-BS*	-1.6±0.1	1.3±1.2	-1.3±1.4
*BBES*	2.0±1.2	2.0±0.2	1.9±1.2
ΔΔCT*_COPD-BS-BBES_*	-3.6	-0.7	-3.2
2^-ΔΔ CT*_COPD-BS-BBES_*	12.1	1.6	9.2

Levels are presented as relative expression measures using the 2^-ΔΔCT method. Medians from three repetitions are presented with standard deviations (SDs).

### Hsp Intracellular Protein Levels

**Figure 1:**
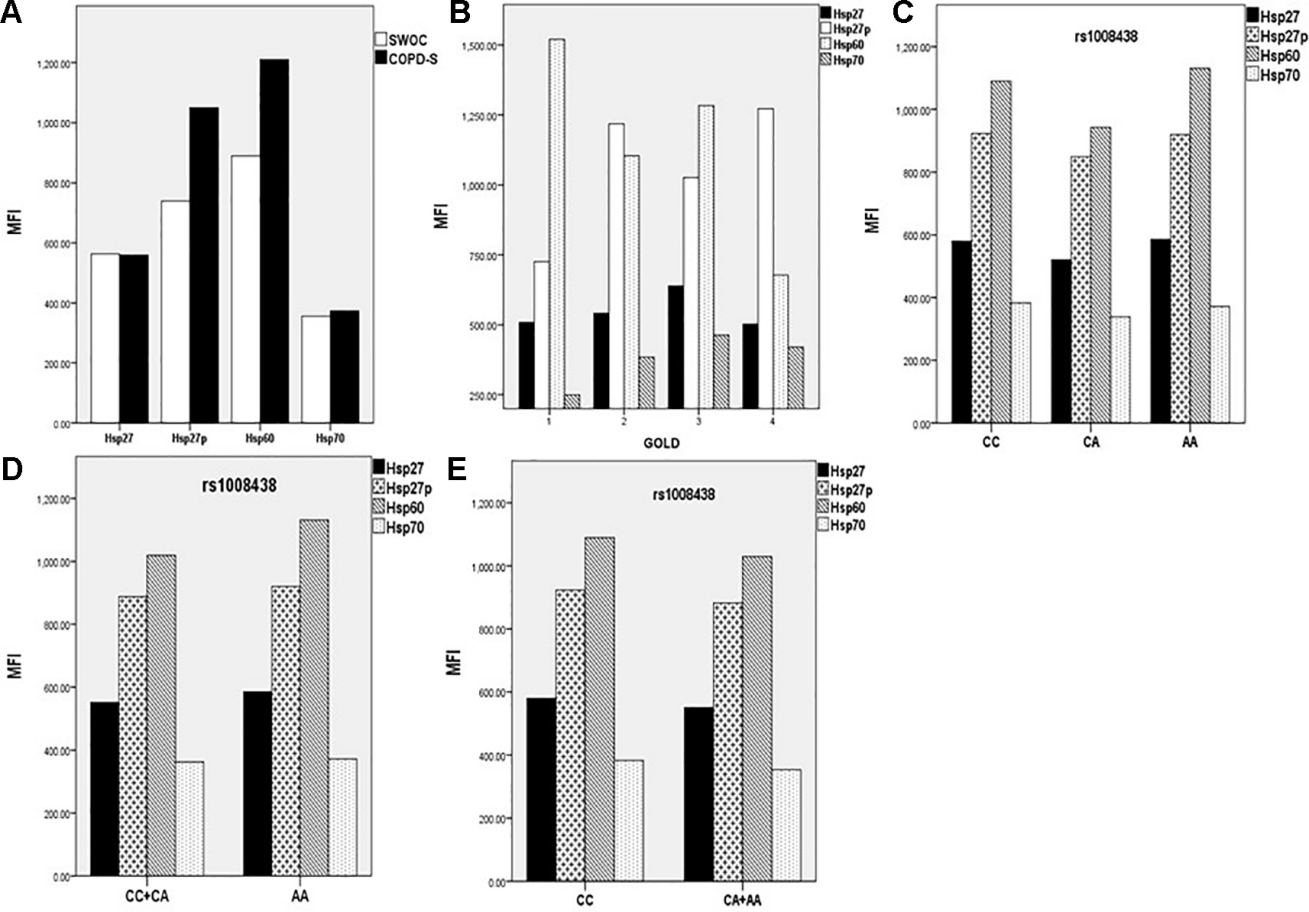
MFI (Median Fluorescent Intensity) comparison between SWOC *vs.* COPD-S for intracellular Hsp. Significant differences in Hsp27p (p=0.01) and Hsp60 (p = 0.025) were observed **(A)**. GOLD stratification comparing Hsp levels **(B)**, stratifying by rs1008438 genotypes **(C)**, recessive model **(D)** and dominant model **(E)**. No significant differences were found for any protein in genotype model analysis.

Samples included in this analysis were chosen according to the findings of genetic associations. We included controls and cases with the three different genotypes of rs1008438 (CC, CA, and AA). When we compared COPD-S *vs.* SWOC, we found higher levels of Hsp27 and Hsp60 in the cases. Next, when the COPD-S group was stratified based on the GOLD severity criteria, we observed increased levels of Hsp60 in GOLD1 compared with the other severity groups; however, this comparison was not significant. As the sample size analyzed was reduced when subjects were stratified by stage of severity, a group of lower severity (G1) (GOLD 1 + GOLD 2) and one of greater severity (G2) (GOLD 3 + GOLD 4) were formed. There were no significant differences between the groups (**Figure 1**).

When we compared BBES *vs.* COPD-BS, we noticed a significant difference in the five protein levels. Next, COPD-BS was stratified by GOLD stage, but no differences in the protein levels were found.

To test the effect of rs10084438 on protein production, we compared Hsp levels under dominant and recessive models. No significant differences were found in any model analyzed.

## Discussion

This is the first study in the Mexican mestizo population to examine the genetic susceptibility between COPD (secondary to tobacco smoking and biomass burning) and HSP family genes, and it is the first to include mRNA levels from induced sputum cells and serum protein levels.

Comparison groups were classified based on tobacco smoking or BBS exposure history. Differences in sex distribution between groups were observed because of their historical and sociocultural backgrounds. It is common in rural regions to find a greater number of people exposed to BBS, and at the same time, this exposed group was women in charge of household chores, among which is cooking, an activity in which they use wood to start and maintain the combustion of biomass to heat food. For that reason, the biomass burning-smoke group had a predominance of females (> 82%). Among the SWOC group, similar proportions of men and women (~50%) were observed, whereas the COPD-S group had a greater percentage of men (> 70%). Most individuals in the control group (SWOC) came from the clinic for the cessation of tobacco smoking; in this group, the distribution was similar between men and women. Most individuals diagnosed with smoking-related COPD are men, probably due to the social taboos in regard to tobacco use by females (Dransfield et al., 2007).

SNPs included in this study were chosen because of the importance of their positions in regulatory regions (rs1008438, rs1043618, rs6457452, rs2763979, and rs17856061) and coding regions (rs562047, rs1061581, and rs2227956) of the HSP genes. These SNPs had been included in other studies focused on the relationship between SNPs and respiratory diseases and cancer, including clinical phenotypes (Partida-Rodríguez et al., 2010; Wang et al., 2010; Berend, 2015) and severity stages (Ding et al., 2011) as well as cellular stress events (Myung et al., 2004; Suzuki et al., 2006; Wang et al., 2010), mainly in Caucasian or Asian populations (Suzuki et al., 2006; Qi et al., 2009; Ding et al., 2011).

HWE deviation is the first quality control in genetic population studies, giving the researchers the option to select a better genetic marker that guarantees the correct selection of subjects. In this study, some of the SNPs with HWE deviation were associated with an increased risk of COPD. In the smoker group, six out of eight SNPs (rs562047, rs1008438, rs6457452, rs2763979, rs17856061, and rs2227956) presented differences (p < 0.05) when cases *vs.* controls were compared, although only rs1008438 and rs562047 followed HWE.

Among those exposed to BBS, we found significant differences in rs1008438 and rs17856061, both of which fulfilled HWE. We must consider that in some populations, HWE is not met because of recombination events. In the Mexican mestizo population, rich genetic variability has been described, a product of the years of genetic recombination between ancestral populations (mainly Amerindian and Caucasian, with a minor contribution African descendants), which could result in some SNPs having a behavior not described in the scientific literature. This situation makes necessary the application of functional/biochemical studies, such as intracellular protein levels or gene expression assays.

Since differences were found in some demographics and clinical variables, it was necessary to adjust by covariables; interestingly, after the analysis, three SNPs (rs562047, rs1008438, and rs2763979) maintained the association, showing that the differences did not interfere with these associations.

In addition, by applying a codominant and a dominant genetic analysis model, rs1008438/A was associated with an increased risk of COPD, both in smokers and in BBS-exposed individuals. On the other hand, when we organized the cases by GOLD stage, in the COPD-S group the same SNP was found to be associated. This SNP had been associated with genetic susceptibility to other lung diseases, such as lung edema (Qi et al., 2009), as well as low Hsp70 levels in granulomatosis (Weingartmann et al., 1999) and with the severity of autoimmune illness (Neef et al., 2011).

The recurrent association of rs1008438 in different types of comparisons suggests that this polymorphism could be a participant in some regulatory mechanisms that have not been described. This information could be a signal that polymorphisms have repercussions in the chronic inflammatory process. Using the data information in HaploReg v. 4.1, we found that the rs1008438 has been associated with two epigenetics changes, one related to transcription activity and one related to an enhance function in lung and T cells (Ward and Kellis, 2012). After QTA, we did not find significant association between the polymorphism rs1008438 and any other in *HSPA1A* (**Supplementary Table 5**). This negative finding could reflect small sample size as well as possible confounding effects of pharmacological treatment.

rs1008438 is in the promoter region of *HSPA1A* and *HSPA1L*. The promoter region in *HSP* genes has highly conserved sequences recognized by specific transcription factors called HSFs (Heat Shock Factor) (Dai et al., 2007; Ciocca et al., 2012; Bei et al., 2013). If this region presents variability in its sequence, this could decrease the mRNA transcription level due to the transcription factors losing binding affinity to the DNA sequence (Dai et al., 2007; Ciocca et al., 2012). When we review the information about rs1008438, in HaploReg v. 4.1, we found a positive alteration in the transcription of *HSPA1A* (Ward and Kellis, 2012). rs1008438/A could promote an alteration in the production capacity of the encoded protein, thereby affecting the inflammatory process. In COPD patients, the presence of CD8+CD28- T-cells has been described, whose principal role is focused on regulating the inflammatory process (Profita et al., 2012; Hodge et al., 2016). It has been shown that in chronic inflammatory events, these cells cannot be depleted by using corticosteroids. This could be explained by the change in the Hsp70 and Hsp90 low expression levels. The receptors for corticosteroids are bound in the cytoplasm to protein chaperone complexes (Hsp70, Hsp90, and Hsp56) (Tissing et al., 2003; Filipović et al., 2005; Bei et al., 2013). When the receptor binds the target molecule, this protein complex dissociates (Gao et al., 2013; Hodge et al., 2016), allowing the translocation of the corticosteroid-receptor complex to the nucleus, where it fulfills its function as a negative regulator in the expression of pro-inflammatory genes (Pratt and Toft, 1997; Hodge et al., 2016). In the absence of these chaperone molecules, the receptor loses affinity and stability, preventing the depletion of CD8+CD28- T-cells or resulting in cell senescence, promoting the inflammatory process. The presence of polymorphisms in the promoter region, such as rs1008438, could explain the resistance and inflammatory dysregulation.

When we analyzed the gene expression of *HSPA1A, HSPA1B,* and *HSPA1L*, we found a decrease in the relative expression levels in the COPD-S group compared with the control group. It has been described that Hsp70 levels increase because of the presence of stress factors, both intrinsic and extrinsic to the patient. Cigarette smoke is widely described as a source of reactive oxygen species (ROS), reactive nitrogen species (RNS), and cytotoxic elements, which are capable of inducing a local inflammatory response (Tsuchiya et al., 2002; Tavilani et al., 2012; Neves et al., 2016). The diminution in *HSPA1A, HSPA1B,* and *HSPA1L* mRNA levels in the cases could have been due to some COPD-S subjects having a pharmacological regimen that included inhaled corticosteroids, promoting the decrease in molecules related to inflammation, Hsp70 among them. On the other hand, most of the people in the SWOC group were current smokers. This continuous interaction between the lung cells and smoke could explain why the mRNA relative expression in control groups was higher than in case groups. Thus, the variation in mRNA relative expression could reflect inhaled corticosteroid medication in the COPD-S group.

In the biomass-smoke exposure comparison, mRNA levels in the three genes were higher in cases than in controls. During the interview of the BBS group, a great proportion of the participants without the illness described discontinuing the use of biomass; in other words, the induction in the expression of these genes had stopped. The case group had higher expression because they suffered an inflammatory process due to biomass smoke, which generates oxidative stress in the environment that alters lung homeostasis.

When Hsp27, Hsp27p, Hsp60, and Hsp70 were measured in the smoker group, we found significant differences in Hsp27p and Hsp60, while among those exposed to biomass smoke, we found increased expression of all proteins. When protein levels were stratified by the genotype of rs1008438, we did not find significant differences. However, stratifying by severity, a trend for an Hsp60 decrease and an increasing tendency for Hsp27p in the smoker group was detected. In the COPD-BS group, no differences were found in the severity stratification. Hsp60 is a pro-inflammatory protein that promotes the production of IL-1β, IL-6, and TNF-α, as well as tumoral-cell survival and nitrogen oxidative species (NOS) production (Caruso Bavisotto et al., 2017; Sangiorgi et al., 2017). At the pulmonary level, Hsp60 is described as an extracellular-stimulation signal for inflammatory activity; Hsp60 is released by epithelial cells when it reaches high levels in response to oxidative stress (Cappello et al., 2006; Sangiorgi et al., 2017). COPD is characterized by the presence of oxidative stress compounds that can promote and maintain the inflammatory response, inducing the production Hsp60. In our study, we found higher levels of intracellular Hsp60 in affected subjects when COPD-S *vs* SWOC were compared, which demonstrates the inflammatory process of the illness and the possible environmental oxidative stress imposed on epithelial cells.

We found that the SNP rs1008438 was associated with COPD but not with *HSPA1A* mRNA or protein levels. This negative finding could be related to pharmacological treatment or small sample sizes. Given the bioinformatic evidence of a possible regulatory effect of rs1008438, we recommend additional study with larger sample sizes.

## Conclusion

rs1008438 (A allele and genotypes containing it) is associated with genetic susceptibility to COPD, both in smokers and in biomass-burning exposed subjects; at the same time, it predisposes smokers to severe forms of COPD, but the presence of this SNP did not significantly affect the Hsp70 protein or mRNA level.

## Data Availability Statement

The datasets generated for this study can be found in ClinVar accessions SCV000999076–SCV000999082.

## Ethics Statement

The studies involving human participants were reviewed and approved by Ethics in Research Committee of the INER in Mexico City (protocol numbers B14-17 and B11-19). The patients/participants provided their written informed consent to participate in this study.

## Author Contributions

EA-O: review of literature, DNA/RNA isolation, development of molecular biology techniques (genotyping, expression assays, protein level determination), bioinformatic analysis, manuscript drafting. GP-R: bioinformatic and statistical analysis. AR-V: enrollment of patients, clinical revision, and statistical analysis. RH-Z: enrollment of patients, clinical revision, and statistical analysis. AA-P: development of molecular biology techniques, bioinformatic analysis. MP-R: development of molecular biology techniques, bioinformatic analysis. AS: development of molecular biology techniques, bioinformatic analysis. EA-R: bioinformatic and statistical analysis. RF-V: development of molecular biology techniques, bioinformatic analysis, manuscript drafting.

## Funding

This work is supported by the allocated budget to research (RFV-HLA Laboratory) from the Instituto Nacional de Enfermedades Respiratorias Ismael Cosío Villegas (INER). Supported partially by the resource assigned to INER, managed in Legislatures of the Chamber of Deputies, through its Committee on Equality and Gender, for the budgetary allocation for the Care of Using Wood Associated Diseases.

## Conflict of Interest

The authors declare that the research was conducted in the absence of any commercial or financial relationships that could be construed as a potential conflict of interest.
