# Supplementary material for: Effect of SNPs in HSP Family Genes, Variation in the mRNA and Intracellular Hsp Levels in COPD Secondary to Tobacco Smoking and Biomass-Burning Smoke
Source: Front Genet. 2020 Jan 9;10:1307. doi: 10.3389/fgene.2019.01307 (PMC6962328; doi:10.3389/fgene.2019.01307)
Supplement: Supplementary file 1 [file DataSheet_1.docx]

Supplementary Material

**Supplementary Table 1. Allele comparison in the smokers’ groups.**

| **Genes/SNP** | **COPD-S** | **SWOC** | **p** | **p_B_** | **OR** | **CI (95%)** |
| --- | --- | --- | --- | --- | --- | --- |
|  | **AF% (n=360)** | **AF% (n=743)** |  |  |  |  |
| *HSPA1A* | | | | | | |
| rs562047 |  |  |  |  |  |  |
| G | 80.31 | 84.91 | <0.01 | <0.01 | 0.72 | (0.73-0.92) |
| C | 19.69 | 15.09 |  |  | 1.38 | (1.09-1.74) |
| rs1008438 |  |  |  |  |  |  |
| C | 51.21 | 58.69 | <0.01 | 0.02 | 0.74 | (0.61-0.89) |
| A | 48.79 | 41.31 |  |  | 1.35 | (1.13-1.63) |
| rs1043618 |  |  |  |  |  |  |
| G | 64.61 | 65.07 | 0.86 | 1.00 | 0.98 | (0.81-1.18) |
| C | 35.39 | 34.93 |  |  | 1.02 | (0.85-1.23) |
| rs1061581 |  |  |  |  |  |  |
| G | 47.27 | 51.25 | 0.09 | 0.17 | 0.85 | (0.71-1.02) |
| A | 52.73 | 48.75 |  |  | 1.17 | (0.98-1.41) |
| *HSPA1B* | | | | | | |
| rs6457452 |  |  |  |  |  |  |
| C | 54.40 | 60.25 | 0.01 | 0.08 | 0.79 | (0.65-0.95) |
| T | 45.60 | 39.75 |  |  | 1.27 | (1.06-1.53) |
| rs2763979 |  |  |  |  |  |  |
| C | 42.68 | 50.21 | <0.01 | <0.01 | 0.74 | (0.62-0.89) |
| T | 57.32 | 49.79 |  |  | 1.35 | (1.13-1.62) |
| *HSPA1L* | | | | | | |
| rs17856061 |  |  |  |  |  |  |
| C | 76.61 | 84.68 | <0.01 | <0.01 | 0.57 | (0.46-0.72) |
| G | 23.39 | 15.32 |  |  | 1.78 | (1.42-2.23) |
| rs2227956 |  |  |  |  |  |  |
| T | 60.83 | 55.43 | <0.01 | <0.01 | 1.36 | (1.13-1.63) |
| G | 39.17 | 44.57 |  |  | 0.74 | (0.61-0.89) |

p values were calculated by Fisher’s exact test. We considered a significant association when the p-value was <0.05. p_B_=Bonferroni correction p-value.

**Supplementary Table 2. Genotype comparison in the smokers’ groups by codominant model.**

| **Gene/SNP** | **COPD-S** | **SWOC** | **p** | **OR** | **CI (95%)** |
| --- | --- | --- | --- | --- | --- |
|  | **AF% (n=360)** | **AF% (n=743)** |  |  |  |
| ***HSPA1A*** |  |  |  |  |  |
| rs562047 |  |  |  |  |  |
| GG | 66.57 | 75.64 |  | 1.00 (Ref) | |
| GC | 27.48 | 18.54 | 0.01 | 1.68 | 1.25-2.28 |
| CC | 5.95 | 5.82 |  | 1.16 | 0.67-2.00 |
| rs1008438 |  |  |  |  |  |
| CC | 25.76 | 34.49 |  | 1.00 (Ref) | |
| CA | 50.91 | 48.40 | <0.01 | 1.41 | 1.04-1.96 |
| AA | 23.33 | 17.11 |  | 1.83 | 1.25-2.66 |
| rs1043618 |  |  |  |  |  |
| GG | 38.48 | 41.62 |  | 1.00 (Ref) | |
| GC | 52.25 | 46.89 | 0.83 | 1.21 | 0.92-1.58 |
| CC | 9.27 | 11.49 |  | 0.87 | 0.56-1.37 |
| rs1061581 |  |  |  |  |  |
| GG | 15.52 | 22.11 |  | 1.00 (Ref) | |
| GA | 63.51 | 58.28 | 0.054 | 1.55 | 1.09-2.20 |
| AA | 20.98 | 19.61 |  | 1.52 | 1.00-2.32 |
| ***HSPA1B*** |  |  |  |  |  |
| rs6457452 |  |  |  |  |  |
| CC | 13.78 | 23.48 |  | 1.00 (Ref) | |
| CT | 81.23 | 73.55 | <0.01 | 1.89 | 1.32-2.68 |
| TT | 4.99 | 2.97 |  | 2.85 | 1.39-5.86 |
| rs2763979 |  |  |  |  |  |
| CC | 14.37 | 21.41 |  | 1.00 (Ref) | |
| CT | 56.62 | 57.60 | <0.01 | 1.46 | 1.02-2.09 |
| TT | 29.01 | 20.99 |  | 2.06 | 1.38-3.08 |
| ***HSPA1L*** |  |  |  |  |  |
| rs17856061 |  |  |  |  |  |
| CC | 58.54 | 72.53 |  | 1.00 (Ref) | |
| CG | 36.13 | 22.53 | <0.01 | 2.93 | 2.19-3.92 |
| GG | 5.32 | 4.95 |  | 1.47 | 0.82-2.61 |
| rs2227956 |  |  |  |  |  |
| TT | 21.67 | 18.46 |  | 1.00 (Ref) | |
| TG | 78.33 | 81.40 | <0.01 | 0.39 | 0.29-0.55 |
| GG | 0.00 | 0.13 |  | ----- | ----- |

p values were calculated by Fisher’s exact test. We considered a significant association when p-value was <0.05.

Supplementary Table 3. Allele comparison in COPD-BS *vs.* BBES group.

| Genes/SNP | COPD-BS  FA% (n=244) | BBES  FA% (n=198) | p | p_B_ | OR | CI (95%) |
| --- | --- | --- | --- | --- | --- | --- |
| *HSPA1A* | | | | | | |
| rs562047 |  |  |  |  |  |  |
| G | 78.22 | 78.5 | 0.98 | 1.00 | 0.98 | (0.71-1.36) |
| C | 21.78 | 21.5 |  |  | 1.02 | (0.74-1.40) |
| rs1008438 |  |  |  |  |  |  |
| C | 54.66 | 63.78 | <0.01 | 0.07 | 0.68 | (0.52-0.90) |
| A | 45.34 | 36.22 |  |  | 1.46 | (1.11-1.92) |
| rs1043618 |  |  |  |  |  |  |
| G | 67.83 | 67.60 | 1.00 | 1.00 | 1.01 | (0.76-1.34) |
| C | 32.17 | 32.40 |  |  | 0.99 | (0.75-1.32) |
| rs1061581 |  |  |  |  |  |  |
| G | 52.04 | 50.52 | <0.01 | 0.03 | 1.06 | (0.81-1.39) |
| A | 47.96 | 49.48 |  |  | 0.94 | (0.72-1.23) |
| *HSPA1B* | | | | | | |
| rs6457452 |  |  |  |  |  |  |
| C | 56.35 | 59.09 | 0.45 | 1.00 | 0.89 | (0.68-1.17) |
| T | 43.65 | 40.91 |  |  | 1.12 | (0.86-1.46) |
| rs2763979 |  |  |  |  |  |  |
| C | 56.10 | 57.87 | 0.65 | 1.00 | 0.94 | (0.70-1.24) |
| T | 43.90 | 42.13 |  |  | 1.07 | (0.83-1.42) |
| *HSPA1L* | | | | | | |
| rs17856061 |  |  |  |  |  |  |
| C | 85.80 | 79.34 | 0.01 | 0.08 | 1.57 | (1.11-2.24) |
| G | 14.20 | 20.66 |  |  | 0.64 | (0.45-0.90) |
| rs2227956 |  |  |  |  |  |  |
| T | 60.67 | 59.95 | 0.89 | 1.00 | 1.03 | (0.78-1.36) |
| G | 39.33 | 40.05 |  |  | 0.97 | (0.74-1.28) |

p values were calculated by Fisher’s exact test. A significant association was considered when the p-value was <0.05. p_B_=Bonferroni correction p-value.

**Supplementary Table 4. Genotype comparison in BBE group by codominant model.**

| **Gen/SNP** | **COPD-BS** | **BBES** | **p** | **OR** | **CI (95%)** |
| --- | --- | --- | --- | --- | --- |
|  | **n=244 (%)** | **n=198 (%)** |  |  |  |
| ***HSPA1A*** |  |  |  |  |  |
| rs1008438 (C/A) |  |  |  |  |  |
| CC | 18.25 | 38.78 | <0.01 | 0.35 | 0.24-0.52 |
| CA+AA | 81.75 | 61.22 |  | 2.84 | 1.92-4.18 |
| rs1043618 (C/G) |  |  |  |  |  |
| GG | 44.67 | 44.90 | 0.96 | 0.99 | 0.68-1.45 |
| GC+CC | 55.33 | 55.10 |  | 1.01 | 0.69-1.47 |
| ***HSPA1L*** |  |  |  |  |  |
| rs17856061 (G/C) |  |  |  |  |  |
| CC | 74.49 | 68.4 | 0.19 | 1.35 | 0.89-2.04 |
| CG+GG | 25.51 | 31.6 |  | 0.74 | 0.49-1.12 |

p values were calculated by Fisher’s exact test. A significant association was considered when the p-value was <0.05. Only SNPs that met HWE were included.

Supplementary Table 5. QTA for *HSPA1A* in smokers and BBES.

| **SNP** | **Beta** | **r^2^** | **t** | **p** |
| --- | --- | --- | --- | --- |
| **Smokers** | | | | |
| rs562047 | -0.9295 | 0.06 | -1.2 | 0.21 |
| rs1008438 | 0.7946 | 0.05 | 1.2 | 0.23 |
| rs1043618 | 0.114 | 0.002 | 0.2 | 0.81 |
| rs1061581 | -0.2791 | 0.008 | -0.5 | 0.63 |
| **BBES** | | | | |
| rs562047 | -0.6 | 0.04 | -1.0 | 0.32 |
| rs1008438 | 0.3174 | 0.02 | 0.7 | 0.49 |
| rs1043618 | -0.04235 | 0.0001 | -0.06 | 0.95 |
| rs1061581 | -0.03889 | 8.84E-05 | -0.04 | 0.96 |

BBES: biomass-burning exposed subjects. t= Wald test value. p= Wald test p value. r^2^: Correlation value between *HSPA1A* and SNP.
